# Empirical Study of Monthly Economic Losses Assessments for “Standard Unit Lockdown” Due to COVID-19

**DOI:** 10.3389/fpubh.2022.859751

**Published:** 2022-05-10

**Authors:** Houli Zhang, Shibing You, Miao Zhang, Anqi Chen, Zengyun Hu, Ying Liu, Difei Liu, Pei Yuan, Yi Tan

**Affiliations:** ^1^School of Economics and Management, Wuhan University, Wuhan, China; ^2^State Key Laboratory of Desert and Oasis Ecology, Xinjiang Institute of Ecology and Geography, Chinese Academy of Sciences, Urumqi, China; ^3^LAMPS and Centre for Diseases Modelling (CDM), York University, Toronto, ON, Canada; ^4^Department of Mathematics and Statistics, York University, Toronto, ON, Canada

**Keywords:** COVID-19, lockdown, standard unit incident, assessment system, monthly economic losses assessment

## Abstract

**Background:**

The pandemic of COVID-19 has been shaping economic developments of the world. From the standpoint of government measures to prevent and control the epidemic, the lockdown was widely used. It is essential to access the economic losses in a lockdown environment which will provide government administration with a necessary reference for decision making in controlling the epidemic.

**Methods:**

We introduce the concept of “standard unit incident” and an economic losses assessment methodology for both the standard and the assessed area. We build a “standard unit lockdown” economic losses assessment system and indicators to estimate the economic losses for the monthly lockdown. Using the comprehensive assessment system, the loss infected coefficient of monthly economic losses during lockdown in the 40 countries has been calculated to assess the economic losses by the entropy weighting method (EWM) with data from the CSMAR database and CDC website.

**Results:**

We observe that countries in North America suffered the most significant economic losses due to the epidemic, followed by South America and Europe, Asia and Africa, and Oceania and Antarctica suffered relatively minor economic losses. The top 10 countries for monthly economic losses during lockdown were the United States, India, Brazil, France, Turkey, Russia, the United Kingdom, Italy, Spain, and Germany. The United States suffered the greatest monthly economic losses under lockdown ($65.3 billion), roughly 1.5 times that of China, while Germany suffered the least ($56.4 billion), roughly 1.3 times that of China.

**Conclusion:**

Lockdown as a control and mitigation strategy has great impact on the economic development and causes huge economic losses. The economic impact due to the pandemic has varied widely among the 40 countries. It will be important to conduct further studies to compare and understand the differences and the reasons behind.

## 1. Introduction

Currently, the COVID-19 has been spreading throughout the world. Waves of outbreaks continue to swape throughout countries worldwide with emerging of new strains of virus. As declared by the World Health Organization (WHO), governments must balance between epidemic prevention and control and economic and social development in the face of this global pandemic. Without the efficient vaccine, non-pharmaceutical interventions (NPIs), including the lockdown, contact tracing and isolation of cases, physical distance are effective mitigation strategies widely applied around the globe. The lockdown has been proven to be the most effective policy for control the spread, however its cost can be enormous. To battle the unprecedented epidemic, public health and governments require a concise, rapid, objective, and scientific basis for making decisions for epidemic prevention and control. Therefore, it is critical to conduct an objective economic assessment of an epidemic prevention and control strategy. The assessment of the economic losses not only provides a basic and realistic understanding of the effectiveness of policy but also supplied a scientific judgment when facing a continuing significant public health event.

Research on the economic losses under public health events has primarily focused on exploratory studies at the micro and macro levels. At the micro-level, the assessment of economic losses under public health events is primarily comprised of two components: epidemic data and a loss evaluation indicator system. The sources of epidemic data are divided into two categories: actual epidemic data and simulation data from the epidemic model simulations, which are used to calculate economic losses from real epidemics and analyze the return on investment (ROI) of prevention and control strategies, respectively. The dynamic transmission models and calibration modeling are the two main areas of micro-level research. The compartment models, network models, metapopulation models, and Agent based simulation models (1) are the popular tools to mimic the transmission of infectious disease. Medical losses, absenteeism losses, disinfection expenditure, and transmitting vector control costs are all factored into the economic damage assessment (2).

At the macro-level, the input-output (IO) method or general equilibrium model (CGE) (3) is the most commonly used approach for assessing economic losses under public health events. These models analyze the impact of an epidemic on individual industry sectors and the economy as a whole. The IO approach is essentially a subset of the CGE model, which is currently the dominant method for calculating losses to economic systems caused by exogenous shocks. CGE models, which use multiple non-linear equations to simulate the behavior of production, consumption, and employment of economic entities in a social-economic system, are a standard tool for estimating aggregate economic losses. In comparison to the IO model, the CGE model reflects the interdependence within the economic system's factors. It overcomes the IO model's shortcomings such as lack of behavioral response, insensitivity to market price changes, and linear, rigid, and inelastic input-output coefficients (4), and constructs a model more in line with reality (5).

The assessment of economic damages for the COVID-19 epidemic belongs to the macro-level, and the IO approach or CGE analysis is more demanding in terms of data requirements, assessment content, and analysis process. Wuhan was the first city to implement lockdown for stamp out COVID-19 in China. You et al. (6) assessed Wuhan's monthly lockdown economic losses using the IO method and the CGE analysis. There are also related studies that have applied event review research methods, vertical and horizontal analysis methods, comparative static analysis methods, and path analysis methods to evaluate the economic losses caused by COVID-19 (7). The available literature provides valuable reference for our study, and this article in the pertinence of the assessment method and the comprehensiveness of the assessment scope have been further improved. Given the Wuhan/Hubei experience, a comprehensive set of interventions, including aggressive case and contact identification, isolation and management and extreme social distancing, had been implemented to interrupt the chains of transmission nationwide (8). Then, the epidemic spread around the world. To date, some countries had also taken a set of interventions like what China did, which means that they had been during lockdown for some times to fight against the epidemic. Given the situation, in this paper we propose a novel method for assessing economic losses using “standard unit incident”, and use the case of Wuhan lockdown as a standard unit for case study to develop a basic reference system for evaluating the monthly economic losses. Furthermore, for the selected 40 countries which implemented lockdown measures, we will use the method to assess and compare their monthly economic losses.

## 2. Methods

### 2.1. Data Resources

The relevant industry and consumption data from each of the 40 countries will be used to assess the economic losses in the event of a lockdown. It includes the data of the percentage of the industrial output, the percentage of construction output, the percentage of wholesale, retail, and catering production of GDP, and the percentage of transportation, storage, and postal and telecommunications output of GDP, the proportion of consumer spending of GDP, the percentage of government final consumption expenditure of GDP, and the percentage of gross fixed capital formation of GDP.

There are more countries applied the lockdown policy. In this paper, we will select 40 countries that have adopted lockdown measures with available data of various indicators in particular, with relatively large economies. The industry and consumer panel data for these 40 countries in 2019 from the China Stock Market & Accounting Research Database(CSMAR database)were used as the economic development in 2019 was unaffected by the epidemic and can reflect the economic scale of each country in regular times. In addition, we obtain the infection related data due to the epidemic of COVID-19 from the CDC website (9) and the World Health Organization (WHO) report on COVID-19 cases (10). We use the epidemic data till Nov 11, 2021. The data of China does not include Taiwan, Hong Kong, and Macau.

### 2.2. Standard Unit Lockdown and Economic Losses Assessment System

Macroeconomic losses assessment uses economic theories and methods to identify, measure, and valuations of losses items, quantities, and amounts arising from natural disasters, public health events, conflicts, and outbreaks of various animal diseases. The most significant difference between the two is that the purpose of the assessment differs from the volume of the object assessed. Macroeconomic losses assessment is typically conducted after a major catastrophic event in a country or region. In order to develop scientific and rational disaster prevention and relief policy, it is necessary to understand the economic losses caused by the event sooner, and economic loss assessment falls under the category of emergency macro information. As a result, one of the most important aspects of a macroeconomic assessment is to select a quick economic losses assessment that is both simple to use and scientifically sound. Based on this background, our team investigates economic losses assessment after an animal blight outbreak and proposes the concept of “standard unit blight”, (11, 12) laying the groundwork for “standard unit incident” and its economic loss assessment methodology. The concept of “standard unit incident” and its economic losses assessment proposed in this paper is part of the more general scope of the methodological system for assessing economic losses.

“Standard unit incident” of public health refers to a representative incident of natural disasters, public health events, conflicts, and animal blights that have occurred throughout human history and for which economic losses have been assessed or economic losses statistics have been compiled. As “standard unit incidents,” such as “standard unit earthquake,” “standard unit epidemic,” “standard unit tsunami,” “standard unit flood,” “standard unit war,” and so on, they constitute a framework of reference for the rapid economic losses assessment for ongoing incidents and serve as the foundation for assessing the economic loss of prevention and control measures.

The most common feature of a “standard unit incident” is its high influential intensity and broad influential area, the dual aspects of the incident's first and general nature, and the incident's historical normalcy. The “standard unit lockdown” and economic loss assessment aims to provide a scientific basis for assessing cost for informing the decision-making on the prevention and control of the COVID-19 epidemic in countries worldwide as the epidemic spreads.

When COVID-19 outbreak first occurred in Wuhan city, the Chinese government imposed an unprecedented lockdown policy to stop its spread. This powerful measure helped curb the epidemic, however the cost and economic losses were enormous. In this paper, we will sue the idea developed in You et al. (6) to conduct the monthly economic losses incurred during the Wuhan lockdown and use the “standard unit lockdown” to estimate the potential monthly economic losses as reference for assessing the economic loss of other epidemic countries implemented lockdown policy.

For a lockdown country or region, we assess the health burden, resident mental health losses, economic losses of directly damaged industries, and indirect economic losses. We do not account for other health impacts or subsequent health losses related to COVID-19.

#### 2.2.1. Indicators for Measuring the Monthly Economic Losses

Wuhan was the first city to implement a lockdown policy to control the COVID-19 outbreak, so the Wuhan “standard unit lockdown” has all the hallmarks of a “standard unit incident.” The following are the principles for the assessment:

The period for assessment is for a period of 1 month. Because the lockdown time varies by country or city, the assessment is based on a monthly time interval for comparative and normative economic analysis.

This paper uses the Wuhan standard unit lockdown economic losses as a benchmark for China's “state-level standard unit” economic losses data by establishing the corresponding assessment indicator system and using the information about Wuhan standard unit lockdown economic losses in the data package, the proportional relationship between Wuhan's economic losses and China's economic losses in 2020, and the proportional relationship between Wuhan's epidemic data and China's epidemic data.

Based on the comprehensive assessment indicator system, the economic losses scores and health economic losses scores of 40 countries will be calculated using the entropy weighting method (EWM) (13), considering the various factors associate with the economic losses. We take the logarithm and adjust the coefficients of the 40 countries' economic losses scores and health economic losses scores derived using the Chinese “state-level standard unit” as the benchmark.

#### 2.2.2. Construction of the Assessment System

The assessment system constructed in this paper focuses on three major aspects as primary assessment variables: economic losses in industry, social-economic and economic losses in population health. Each primary assessment variable contains several secondary assessment variables.

As a result of the “lockdown,” the urban transportation industry was utterly shut down. The transportation industry is intertwined with many other industries, including warehousing and retail, vacation tourism, lodging and catering, film and cultural industries, etc. Furthermore, the real estate sector was halted, as were the financial and construction industries and other related upstream and downstream industries, all of which were directly or indirectly impacted. Therefore, four indicators were used to reflect the economic losses: percentage of the industrial output of GDP, the percentage of construction output of GDP, the percentage of wholesale, retail, and catering production of GDP, and the percentage of transportation, storage, and postal and telecommunications output of GDP.

The “lockdown” has also affected the various economic entities' consumption level and capital accumulation speed. As the population's purchasing power falls below what it would have been in regular times, government expenditures fall in most areas, except those related to epidemic prevention, and the speed of capital accumulation slows. Therefore, in this paper we use three indicators to reflect social-economic losses: the percentage of consumer spending, government final consumption expenditure, and gross fixed capital formation of GDP.

The number of cures, deaths, and infections associated with the COVID-19 epidemic were chosen as specific variables to measure the health economic losses. We consider the indicators of health and economic losses in the evaluation system is because these people directly related to COVID-19, their cures, deaths due to COVID-19, The inability to participate in social work normally after infection will consume medical costs and social resources.

These variables summarized in Table 1 which include economic indicators for industries, social-economic indicators reflecting government purchases, consumer spending, capital accumulation, and epidemic indicators that can reflect specific COVID-19 infections. This assessment indicator system provides a comprehensive picture of the industry's economic and social-economic losses and the health economic losses.

**Table 1:** Comprehensive assessment indicator system for monthly economic losses of COVID-19.

**General objectives**	**Primary assessment variables**	**Secondary assessment variables**
Comprehensive assessment indicator system for monthly economic losses of COVID-19	Industry's economic losses	The percentage of the industrial output of GDP (%)
		The percentage of the construction output of GDP (%)
		The percentage of the wholesale, retail and catering output of GDP (%)
		The percentage of the transport, storage and post and telecommunications output of GDP (%)
	Social-economic losses	The percentage of consumer spending of GDP (%)
		The percentage of government final consumption expenditure of GDP (%)
		The percentage of gross fixed capital formation of GDP (%)
	Health economic losses	Cures (person)
		Deaths (person)
		Infections (person)

#### 2.2.3. Data Processing and Calculation of Assessment Variables

Due to the different units of variables, the EWM was used twice in this study. At the first time, 40 (m) assessment objects and 10 (n) assessment variables are used to assess the monthly lockdown economic losses (14). First, a raw data matrix X was created based on the research question and statistical data as


(2.2.1) $$\begin{array}{l} X=\left(\begin{array}{cc} x_{11} & x_{1n}\\ x_{m1} & x_{mn} \end{array}\right) \end{array}$$

To obtain the standardized score matrix for each variable, we normalized the raw data matrix X. We use the extremum method to mormalize the raw data, which can be divided into three categories of variables based on the target orientation: positive variables, inverse variables, and neutral variables, which are treated in the following manner.

The falling semi-trapezoid distribution function is applied to calculate positive variables, that is:


(2.2.2) $$\begin{array}{l} r_{ij}=\{\begin{array}{ll} 0 & ,\left(x_{\text{ij}}\leq a_{\min}\right)\\ \left(x_{ij}-a_{\min}\right)/\left(a_{\max}-a_{\min}\right) & ,\left(a_{\min}\leq x_{\text{ij}}\leq a_{\max}\right)\\ 1 & ,\left(x_{\text{ij}}\geq a_{\max}\right) \end{array} \end{array}$$

The triangular distribution function is applied to calculate neutral variables, that is:


(2.2.3) $$\begin{array}{l} r_{ij}=\{\begin{array}{ll} \left(x_{ij}-a_{\min}\right)/\left(a-a_{\min}\right) & ,\left(a_{\min}<x_{\text{ij}}\leq a\right)\\ \left(a_{\max}-x_{ij}\right)/\left(a_{\max}-a\right) & ,\left(a<x_{\text{ij}}<a_{\max}\right)\\ 0 & ,\left(x_{\text{ij}}\geq a_{\max}orx_{\text{ij}}\leq a_{\min}\right) \end{array} \end{array}$$

The ascending semi-trapezoid distribution function is applied to calculate inverse variables, that is:


(2.2.4) $$\begin{array}{l} r_{ij}=\{\begin{array}{ll} 0 & ,\left(x_{\text{ij}}\geq a_{\min}\right)\\ \left(a_{\max}-x_{ij}\right)/\left(a_{\max}-a_{\min}\right) & ,\left(a_{\min}<x_{\text{ij}}<a_{\max}\right)\\ 1 & ,\left(x_{\text{ij}}\leq a_{\max}\right) \end{array} \end{array}$$

The two variables of number of deaths and infections are positive in the evaluation system, implying that the higher the value of the variable, the greater the economic loss. In contrast, the eight variables of the percentage of the industrial output of GDP, the percentage of construction output of GDP, the percentage of wholesale, retail, and catering production of GDP, and the percentage of transportation, storage, and postal and telecommunications output of GDP, the percentage of consumer spending of GDP, the percentage of government final consumption expenditure of GDP, and the percentage of gross fixed capital formation of GDP, the number of cures are inverse variables, implying that the higher the value of the variable, the lower the economic loss.

The standardized matrix is as follows:


$$R={\left(r_{ij}\right)}_{m\times n}$$

where *r*_*ij*_ is the standardized value of the i-th evaluation object on the j-th evaluation variable in the above equation, and


(2.2.5) $$\begin{array}{l} r_{ij}\in \left[0,1\right]. \end{array}$$

The entropy value of an evaluation variable is determined by the definition of entropy when there are m evaluation objects and n evaluation variables. The following is the calculation formula:


(2.2.6) $$\begin{array}{l} E_{j}=-\frac{1}{\ln m}\times \overset{m}{\underset{i=1}{\sum }}f_{ij}\times \ln f_{ij} \end{array}$$

where


(2.2.7) $$\begin{array}{l} f_{ij}=r_{ij}/\overset{m}{\underset{i=1}{\sum }}r_{ij},\left(i=1,2,\cdots \;,m;j=1,2,\cdots n\right) \end{array}$$

But, when *f*_*ij*_ = 0, the logarithm ln *f*_*ij*_ has no meaning. Hence, we use the modified *f*_*ij*_:


(2.2.8) $$\begin{array}{l} f_{ij}=\left(r_{ij}+1\right)/\overset{m}{\underset{i=1}{\sum }}\left(r_{ij}+1\right),\left(i=1,2,\cdots \;,m;j=1,2,\cdots n\right) \end{array}$$

Therefore, the calculation formula *W*_*j*_ (the entropy weight of the j-th assessment variable) is as follows:


(2.2.9) $$\begin{array}{l} W_{j}=\left(1-E_{j}\right)/\overset{n}{\underset{j=1}{\sum }}\left(1-E_{j}\right) \end{array}$$

#### 2.2.4. Classification of Criteria for Economic Losses of COVID-19

According to China State Contingency Plan for Rapid Response to Public Emergencies (15) and State Contingency Plan for Rapid Response to Work Safety Accidents (16), there are four levels of accident response: severe accident (Level I), serious accident (Level II), larger accident (Level III), and ordinary accident (Level IV).

In this regard, variable scores indicate the severity of economic losses during COVID-19 in different countries. Higher variable scores indicate a higher level of economic losses in that country and vice versa. Table 3 presents the range of variable scores for each risk level of economic losses of COVID-19.

In order to visualize and compare the severity of the monthly economic losses of the epidemic in different countries, we will apply the equal difference categorizing method in the classified statistic graph based on the actual data to grade the monthly economic losses scores of 40 countries, as shown in Table 2, we will use the four levels of risk to quantify the corresponding losses of severity from the level of ordinary, high, serious and severe.

**Table 2:** Criteria for economic losses of COVID-19.

**Risk level of COVID-19**	**The meaning of risk level**	**Range of variable scores**
**Level I**	Severe	6.5<
**Level II**	Serious	5.5<≤6.5
**Level III**	High	4.5<≤5.5
**Level IV**	Ordinary	3.5<≤4.5

## 3. Results

Any event will result in an iconic benchmark. The COVID-19 outbreak was first reported in Wuhan, and the Chinese government responded with the first “lockdown”, for the purpose of curbing the epidemic. The evaluation methods and results of this historic lockdown economic losses would serve as a standard reference. We will use Wuhan as a standard unit to study the economic losess during lockdown.

For the assessment of losses for lockdown, we first obtained the total monthly economic losses for Wuhan lockdown which was $25.39 billion. Direct economic losses in the directly affected sectors (transportation, logistics and storage, postal service, hospitality, and catering) totaled $3.111 billion, with indirect economic losses totaling $5.22 billion. The total health economic losses was $17.071 billion. It includes population health burden losses and population mental health economic losses. The former one amounts to $644 million, and the later one amounts to $15.427 billion.

**Figure 1:**
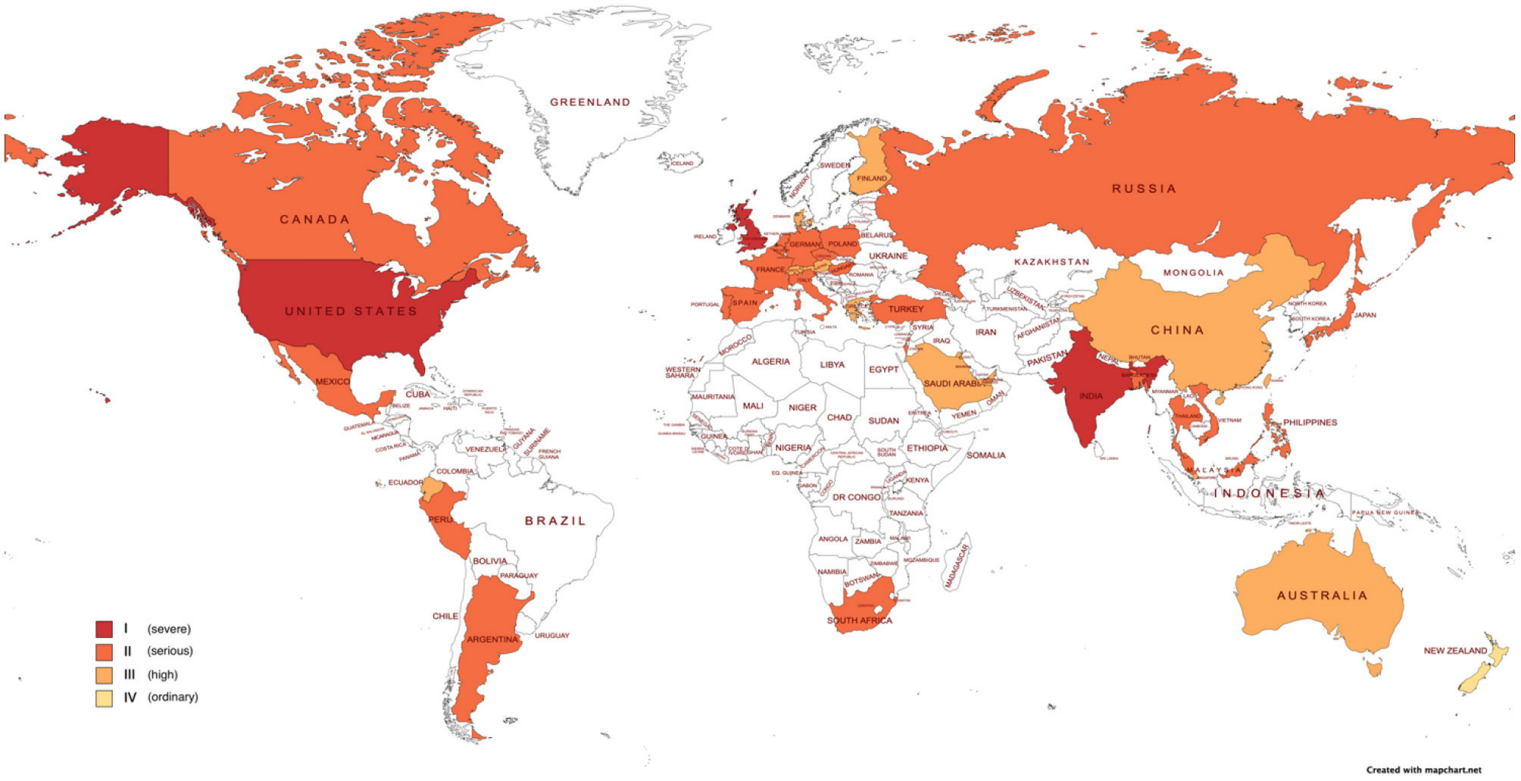
Each country's monthly lockdown economic losses.

We use the concept of “standard unit incident” and an economic losses assessment methodology developed for Wuhan as a standard area and then apply to other areas and countries. We estimate the monthly economic losses of the 40 countries by using adjustment coefficients associate with the study area. The details are shown in Figure 1.

Meanwhile, according to the criteria for economic losses of COVID-19 and each country's score, the United States, India, and United Kingdom are classified as Level I; France, Turkey, and 24 other countries are classified as Level II; Austria, Switzerland, and 12 other countries are classified as Level III; New Zealand is classified as Level IV.

**Figure 2:**
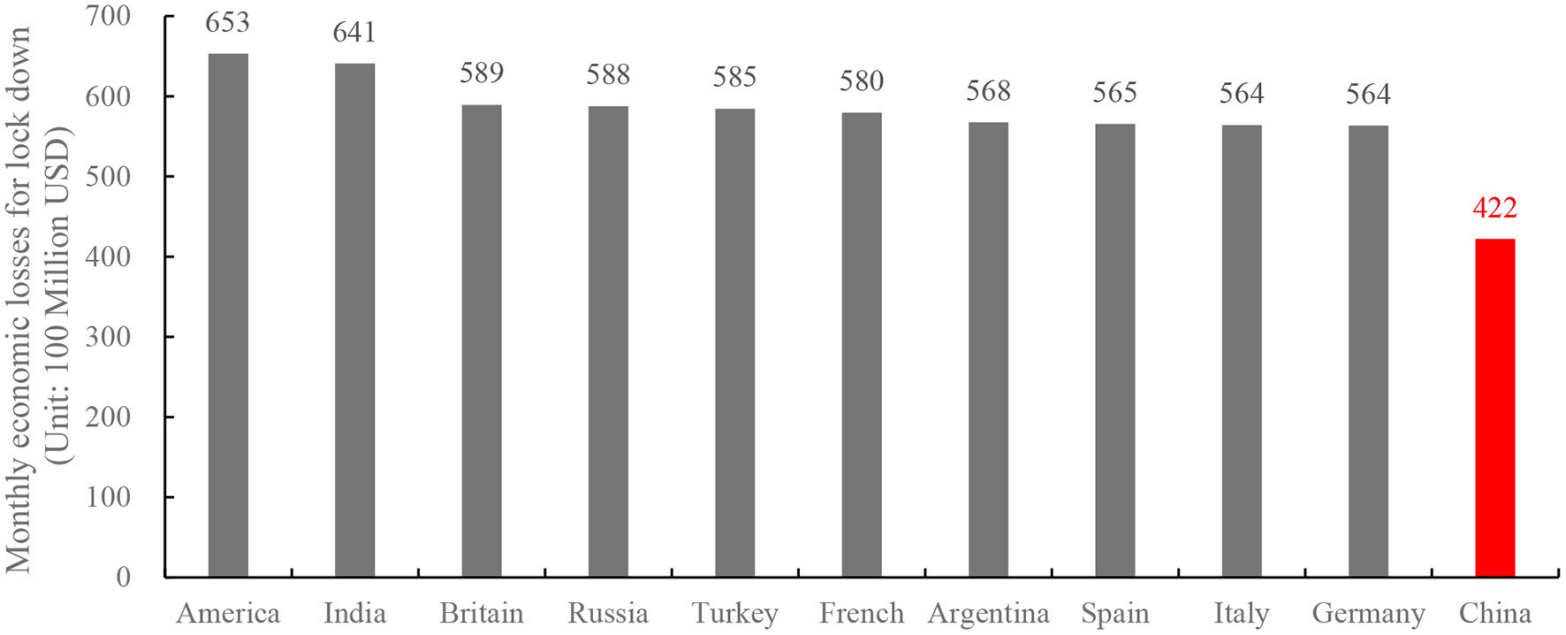
Monthly economic losses for lockdown (unit: 100million).

As shown in Figure 2, North America suffered the greatest economic losses from the epidemic, followed by Asia and Europe, South America and Africa, and Oceania and Antarctica, with Oceania and Antarctica suffering relatively low economic losses.

Using the Wuhan “standard unit lockdown” data package as a reference, the economic losses for China's lockdown were calculated, and the monthly economic losses for each assessed 40 countries were calculated (see Supplementary Material). The top ten countries in terms of monthly economic losses during lockdown are the United States, India, the United Kingdom, Russia, Turkey, France, Argentina, Spain, Italy and Germany. Among them, the United States suffered the greatest monthly economic losses during lockdown, roughly 1.5 times that of China, while Germany suffered the least, roughly 1.3 times that of China. The top ten countries' average monthly economic losses for lockdown were $58.97 billion. Figure 2 depicts the precise amount of losses.

We use EWM to estimate the monthly economic losses for the 40 countries that have adopted the lockdown policy by taking the economic loss of Wuhan for 1 month as the standard unit. As the total economic volume and level of economic development in various countries are different, the relative impact of equivalent absolute economic losses on the economies of various countries is also different. Therefore, we compare the estimated monthly economic losses of 40 countries using their monthly GDP in 2020, and obtains the loss infected coefficients of influence of the monthly economic losses of the lockdown countries.

These coefficients take into account the economic levels of different countries, reflects the loss of COVID-19 associate to GDP, and indicates the extent to which the monthly economic losses of different countries. The loss infected coefficients are presented in Table 3.

**Table 3:** The impact coefficients of monthly economic losses during lockdown in the 40 countries.

	**Country**	**Loss infected coefficient**		**Country**	**Loss infected coefficient**
1	Ecuador	5.32	21	Thailand	1.16
2	Kuwait	3.92	22	Belgium	1.12
3	Hungary	3.37	23	Poland	1.02
4	Peru	2.63	24	Turkey	0.87
5	Greece	2.57	25	Switzerland	0.81
6	Portugal	2.43	26	Saudi Arabia	0.73
7	Czech Republic	2.43	27	Netherlands	0.67
8	Vietnam	2.13	28	Mexico	0.49
9	Bangladesh	1.82	29	Spain	0.41
10	Finland	1.78	30	Russia	0.39
11	New Zealand	1.70	31	Australia	0.31
12	South Africa	1.69	32	Canada	0.31
13	Philippines	1.67	33	Italy	0.29
14	Malaysia	1.61	34	United Kingdom	0.23
15	Denmark	1.53	35	India	0.22
16	Israel	1.52	36	France	0.22
17	Singapore	1.35	37	Germany	0.15
18	Argentina	1.32	38	Japan	0.11
19	United Arab Emirates	1.31	39	United States	0.04
20	Austria	1.24	40	China	0.03

According to the values of the impact coefficients, we divide the above-mentioned countries into three categories: severe impact; moderate impact; mild controllable impact. The top eight countries with a large impact coefficient: Ecuador, Kuwait, Hungary, Peru, Greece, Portugal, the Czech Republic, and Vietnam are more affected by the economic losses during the lockdown. First of all, the economic aggregates of the above-mentioned countries are relatively small. Second, in the economic loss assessment, the number of infections and deaths are the most weighted, while the death toll and the number of infections in the above-mentioned countries are relatively large. Take Peru and the Czech Republic as examples. The total number of infections and deaths in the two countries are 2.4 and 1.8 million respectively. The resulting high medical costs and loss of human capital have thus increased the economic loss, which has a serious impact on the country's economic growth.

Countries with an impact coefficient of 0.5–2 are mainly moderately developed or developing countries. Their economies volume are relatively large. At the same time, the total number of infections and deaths caused by the epidemic are at a moderate level, therefore their economic development was moderately affected. The rest of the 13 countries with an impact coefficient of 0-0.5, including China, the United States, Japan, Germany, France, India, the United Kingdom, Italy, Canada, Australia, Russia, etc. The above-mentioned countries are mainly developed countries and some in the rapid developing stage, their economic volume is at the fore-front of 40 countries. Therefore, although the total number of infections and deaths such as the United States, India, the United Kingdom, and Russia are among the top four in the world, due to their high level of economic development, the economy The volume is large, and the impact of economic losses from the lockdown on economic growth is slightly controllable. It is worth noting that, according to the monthly economic losses from the lockdown of cities estimated in this article, the monthly economic losses in China and the United States account for only 3 and 4% of the monthly GDP of these two countries. China's economic growth rate in 2020 will drop by about 3% year-on-year, while the United States' economic growth will shrink by 3.5% in 2020. Therefore, the evaluation results of this article are reliable. In addition, the monthly economic losses of the monthly GDP is 0.5% higher than the percentage GDP reduction in the United States, the loss infected coefficient and percentage GDP reduction in China are the same. Owing to the fact that the number of confirmed cases and deaths during the outbreak in the US was far larger than in China, the consequent Health-related Economic Losses are greater than the percentage GDP reduction. For Thailand and Argentina, which have similar loss infected coefficients, Thailand's economic growth rate in 2020 fell by approximately 6.2% and Argentina's economic growth rate in 2020 fell by approximately 9.9%, while our estimated monthly economic losses for these two countries are 9.6% and 11% of their respective countries' monthly GDP, respectively. Unlike Argentina, Thailand's core business, tourism, was hurt harder during the lockdown, demonstrating that the economic losses estimated by the loss infected coefficient for each country are more comprehensive and reasonable.

## 4. Discussions

By exploring the theory and application of sustainable economic and social economical development, the concept and methodology of the economic losses assessment using “standard unit incidents” serve as a powerful tool: First, economic losses assessments have been developed as various disastrous events such as natural disasters, public health events, and animal blights occurred in human history. For a typical outbreak of any disastrous events, we can quickly assess the economic losses for policy makers to develop and formulate optimal prevention and control strategies. Secondly, various incidents inevitably occur in the course of human economic and social development. When it happens, the most critical and difficult questions for governments is to decide the priority of economy and life. The rapid assessment method of “standard unit incidents” can provide the government with a “governmental morality” coordinate for decision making.

The selection and differences in epidemic prevention and control strategies are worth thinking about and debating. Bo Peng previously claimed that Western countries had better anti-pandemic efforts than China, but CNN recently reported that China was the first to control the Delta virus successfully. Based on the various evaluations in the international community, this paper draws more specific conclusions based on objective data from countries around the world and an assessment of the “standard unit lockdown” methodology. Different countries take different epidemic prevention and control strategies due to differences in their government management systems, resource endowments, cultural traditions, religious beliefs, and political and economic models. The following are the main points:

First, the institutional structure is dominated by two major models: a high degree of unified command and a relatively independent response to epidemics. The former is advantageous to coordinated control mechanisms, effectively containing the spread of the epidemic and ensuring coordinated socioeconomic operations; the latter is advantageous to relatively flexible and differential responses to epidemics in different regions but affects the consistency of coordination in epidemic prevention and control.

Second, the specific measures and requirements for implementing lockdown in various countries were primarily divided into strict lockdown and semi-lockdown, with significant differences in the degree of lockdown. Wuhan, China's first city to implement a lockdown policy, activated the “pause button” mode, halting almost all economic activities. During the first few days of the lockdown, one person per household was allowed to go shopping outside the community every 3 days, a strict foot ban was soon implemented, and community volunteers delivered all household goods. On the other hand, other countries have adopted a semi-lockdown measurement, with some issuing foot-bans and customs-closure that restrict certain areas for activity. The former's strict lockdown policy had a significant economic impact. With the enormous costs of prevention and control, Wuhan generated nearly no revenue during this period. However, the epidemic was curbed quickly and effectively, providing a reliable guarantee for the subsequent resumption of economic activities (17). The latter, with its semi-lockdown, although it ensured the operation of some economic activities and granted a certain degree of residential freedom, was not conducive to the control of the epidemic, increased its spread, and slowed down the process of economic recovery.

Third, there is a deep gap in the results of lockdown measures in countries. Based on the earliest epidemic-stricken 25 countries' epidemiological data (up to the end of 2020), our paper examines the effectiveness of national control measures on the spread of COVID-19. We conclude that strict restrictive policies (lockdown, school closures, cluster restrictions) were significantly effective in controlling the epidemic's spread. A quick and robust government response significantly accelerates the turning point arrival and decreases infections and deaths. During the first wave of the epidemic, China and South Korea had the most successful response strategies. The study found that if China (or Korea) take timely efficient strategy, the other 23 countries would have seen an average reduction of 91 percent (89 percent) in cases and an average reduction of 88 percent (86 percent) in deaths (18). Around the second wave, most countries' responses were significantly weaker than in the first wave, resulting in an average duration of the second wave of the outbreak that was more than twice as long as the first. In the 17 European and American countries, the government's restrictive policies, economic aid (such as financial subsidies, debt relief) were effective in alleviating the epidemic spread, while transportation mobility contributed significantly to the epidemic spread. Simultaneously, the effects of the same policy differ across countries. In Europe and America, Germany, Sweden, France, Spain, and Turkey were the top five most effective countries for restrictive policies. The policies are approximately twice as effective in controlling the spread of the epidemic in Asian and Oceanian countries as they are in Europe and the United States. However, economic aid and health policies (testing policy, mask level requirements, medical resources, contact tracing) have had no discernible impact. Although the epidemic is not over, it is worthwhile to consider and research what scientific control strategies we should employ in the face of the epidemic.

From the time of relaxation, Table 4 collates the periodicity of blockades and the length of blockades studied in this paper as a way of illustrating the blockade policies of the 40 countries. It is important to note that the monthly economic losses of the different countries studied in this paper are measured in months, while Table 4 shows the actual length of the blockade in each country, and therefore does not directly correlate with the monthly economic losses in the previous section. This is an issue that requires further research.

**Table 4:** Each country's lockdown time.

**Country**	**Lockdown periods**	**Length of lockdown (Unit: Day)**
Ecuador	2020.3.15-2020.5.4	51
Kuwait	2020.3.12-2020.3.29 2020.5.10-5.30	39
Hungary	2020.11.11-2021.3.15	125
Peru	2021.1.15-2021.2.14	31
Greece	2020.3.23-2020.5.4 2020.11.7-2020.12.14	81
Portugal	2020.3.8-2020.5.2 2020.11.14	56
Czech Republic	2020.3.16-2020.3.24 2020.10.22-2020.11.20 2021.3.1-2021.3.21	60
Vietnam	2021.7.23-2021.8.6	15
Bangladesh	2021.4.5-2021.4.11 2021.6.28-2021.7.4 2021.7.23-2021.7.13	28
Finland	2021.3.8-2021.3.28	21
New Zealand	2021.8.17-2021.8.23 2021.9.19-2021.9.21	10
South Africa	2020.3.26-2020.4.16	21
Philippines	2020.3.15-2020.5.15	62
Malaysia	2020.3.18-2020.3.31 2021.6.1-2021.6.14	28
Denmark	2020.3.12-2020.4.6	25
Israel	2020.9.18-2020.10.14 2020.3.20-2020.5.15 2020.12.27-2021.1.11	100
Singapore	2020.4.7-2020.6.1 2021.5.16-2021.6.16	85
Argentina	2020.3.20-2020.5.24	66
United Arab Emirates	2020.3.26-2020.5.13	49
Austria	2020.3.16-2020.4.14 2020.11.3-2020.12.7 2020.12.26-2021.2.7	109
Thailand	2020.4.9-2020.5.17 2021.6.28-2021.7.28	69
Belgium	2020.3.13-2020.4.3 2020.11.2-2020.12.13	64
Poland	2020.11.7-2020.11.29 2021.3.20-2021.4.18	53
Turkey	2020.4.23-2020.4.26 2021.4.29-2021.5.17	23
Switzerland	2020.3.16-2020.5.27	73
Saudi Arabia	2020.3.25-2020.9.15	175
Netherlands	2020.10.14-2020.11.4 2020.12.16-2021.3.15	118
Mexico	2020.3.30-2020.6.1 2020.9.18-2020.10.21	98
Spain	2020.3.16-2020.5.2	48
Russia	2020.3.28-2020.4.30 2021.10.28-2021.11.7	45
Australia	2020.3.23-2020.4.27 2020.7.8-2020.9.13 2021.6.26-2021.7.9	118
Canada	2020.3.18-2020.7.31 2020.11.23-2021.3.9	243
Italy	2020.3.10-2020.5.4 2020.10.24-2020.11.30 2021.3.15-2021.4.2	113
United Kingdom	2020.3.23-2020.6.1 2020.11.5-2020.12.2 2021.1.5-2021.2.15	141
India	2020.3.25-2020.5.3 2021.4.19-2021.5.31	83
France	2020.3.17-2020.5.11 2020.10.30-2020.12.1 2021.4.3-2021.5.3	121
Germany	2020.11.2-2020.12.20 2021.3.23-2021.4.18	76
Japan	2020.4.8-2020.5.25	47
United States	2020.3.16-2020.4.7	23
China (Wuhan)	2020.1.23-2020.4.8	76

The timing of countries implementing lockdown measure is very close. China was the first country to experience an outbreak of COVID-19, and it was the first to implement the lockdown policy. Except for Germany and Denmark, other countries began the lockdown in March and April 2020, with the length ranging from one to 3 months, with Denmark being the first and only lockdown. Several countries experienced a second lockdown between July and November 2020 due to the multiple waves of the outbreak all of which lasted approximately 1 month, and the third lockdown in April 2021 lasted also approximately 1 month. As a country with three times of lockdown, Israel's third lockdown occurred on a different timeline than the others and was due to the second outbreak.

We conclude that depending on the economic and epidemiological conditions, the timing of the lockdown and the economic losses will have different outcomes. Although this point is not addressed in this paper at this time, we believe it is an important and worthwhile topic to investigate.

As the epidemic worsens and the more transmissible variants keep emerging and pose new threat to control the epidemic, we must unavoidably to consider the impact of vaccination and economic losses caused by the epidemic.

As we can see from the above epidemic economic losses assessment, the proportion of health economic losses caused by the epidemic is significant. Increasing vaccination rates is the most effective way to reduce health economic losses caused by the epidemic. To that end, we must address several issues:

First, we need to understand the vaccination coverage and epidemic control. There are currently differences in residents' willingness to get vaccinated in countries worldwide: some are actively vaccinating, others are on the fence and hesitant, and others are paralyzed and sluggish about epidemic prevention and control (19). Government research, planning, and epidemiological studies are required to address both the fear of vaccine shortage and vaccine effectiveness. The primary issue confronting humanity today in terms of economic and social development and human health is epidemic control. Governments should put advocacy interventions and policy measures as a priority.

The second is the relationship between vaccination rates and economic health and economic activity losses. In terms of global economic integration, vaccination rates should be considered not just for a single country but for the entire world's population. The uneven development of the global economy and the vast difference in vaccination rates between developed and developing countries will have a significant impact on the global economy's recovery. As a result, international organizations and governments should continuously promote equitable vaccine distribution and improve vaccine supply capacity. International organizations and governments should work together to promote equitable and reasonable vaccine distribution and to continuously improve vaccine supply capacity to aid in the fight against epidemics around the world, particularly in developing countries, as this is an effective way to reduce health economic losses and accelerate global economic recovery.

The third point is about the vaccine effectiveness ratio. We should consider the cost of vaccines and their contribution to economic development. It should be noted that the cost of vaccine development and the economy's sustainable development are two sides of the same coin. On the one hand, vaccine research and development, production, and vaccination process are prohibitively expensive. In China, the R&D and production costs for each vaccine are approximately US$28.68, and with two doses required per person, the cost of universal vaccination for China's 1.4 billion population is approximately 1.4 billion x 2 x 28.68 = US$80.311 billion. In contrast, the national per capita medical cost for confirmed and suspected patients with COVID-19 is approximately US$2,438. In comparison, the vaccine costs much less than the medical costs for sick patients. On the other hand, on an economic and health level, universal vaccinating benefits the population's health and society's economic reboot. It can be observed that any country that vaccinates a large scale of population will be able to quickly break free from the grip of the epidemic and gain a good position in the global economic recovery and development as soon as possible. Thus, spending on universal vaccination is a strategic national investment rather than a pure consumer one, and it is the most efficient way to promote economic recovery, which will have a significant economic and social impact.

The purpose of this paper is to a new methodology for calculating economic losses using a “standard unit event.” This rapid economic losses assessment method is intended to provide a macro-level foundation for decision-making, but it cannot accurately measure the total economic loss during by the lockdown. This is because the economic losses for the lockdown are multifaceted. First, the epidemic impacts the health of the population, and the burden of disease on the population reduces labor productivity, resulting in varying degrees of economic losses in every industry that requires human capital. Second, the epidemic raises public health expenditures and creates a high demand for medical supplies, but this is not considered into loss range in this paper's analysis, which only considers losses to directly affected industries and indirect economic losses to all industries, as well as health economic losses. Third, our research subject is assessing economic losses, and the epidemic's economic impact is not included in the assessment. The epidemic has had a huge impact on all aspects of the economy. Moreover, many countries around the world experienced other nature disasters when batting with COVID-19 such as floods, earthquakes, and animals' blights. We believe that assessing economic losses caused by the superimposed impact of multiple events is an important research question addressed in future research.

## Data Availability Statement

The original contributions presented in the study are included in the article/Supplementary Material, further inquiries can be directed to the corresponding author.

## Author Contributions

SY and HZ designed research. HZ and MZ collected data. ZH, AC, DL, YL, PY, and YT analyzed the results. SY, HZ, and MZ wrote the manuscript. ZH revised the manuscript. All authors read and approved the final manuscript.

## Conflict of Interest

The authors declare that the research was conducted in the absence of any commercial or financial relationships that could be construed as a potential conflict of interest.

## Publisher's Note

All claims expressed in this article are solely those of the authors and do not necessarily represent those of their affiliated organizations, or those of the publisher, the editors and the reviewers. Any product that may be evaluated in this article, or claim that may be made by its manufacturer, is not guaranteed or endorsed by the publisher.
