# Supplementary material for: Empirical Study of Monthly Economic Losses Assessments for “Standard Unit Lockdown” Due to COVID-19
Source: Front Public Health. 2022 May 10;10:859751. doi: 10.3389/fpubh.2022.859751 (PMC9129268; doi:10.3389/fpubh.2022.859751)
Supplement: Supplementary file 1 [file Table_1.DOCX]

**Appendix 1**

**Each Country’s Lockdown Monthly Economic Losses(Unit: US$100 million)**

| Country | Lockdown Monthly Economic Losses | Country | Lockdown Monthly Economic Losses |
| --- | --- | --- | --- |
| United States | 653.33 | Japan | 523.20 |
| India | 640.95 | Bengal | 519.78 |
| United Kingdom | 589.35 | Belgium | 515.51 |
| Russia | 587.83 | Israel | 512.99 |
| Turkey | 584.51 | Portugal | 505.65 |
| France | 579.65 | Vietnam | 501.11 |
| Argentina | 567.69 | Hungary | 498.77 |
| Spain | 565.46 | Austria | 497.28 |
| Italy | 564.03 | Switzerland | 497.11 |
| Germany | 563.53 | Greece | 492.89 |
| Mexico | 556.48 | United Arab Emirates | 489.75 |
| Poland | 546.74 | Saudi Arabia | 478.45 |
| South Africa | 544.60 | Ecuador | 477.69 |
| the Philippines | 542.49 | Kuwait | 466.93 |
| Malaysia | 537.9 | Denmark | 466.15 |
| Peru | 535.27 | Singapore | 441.74 |
| Netherlands | 532.80 | Australia | 434.86 |
| Thailand | 528.46 | Finland | 430.04 |
| Czech Republic | 525.44 | China | 422.10 |
| Canada | 523.53 | New Zealand | 319.07 |
